# Supplementary material for: Contribution of sociodemographic determinants in explaining the nutritional gap between the richest-poorest women of Bangladesh: A decomposition approach
Source: PLoS One. 2022 Sep 1;17(9):e0273833. doi: 10.1371/journal.pone.0273833 (PMC9436068; doi:10.1371/journal.pone.0273833)
Supplement: Attachment — Submitted filename: Plos One.docx [file pone.0273833.s001.docx]

This is an interesting piece of research. Inequality exist everywhere in Bangladesh which slows down the progress in every aspect in the country. This research in important to address the inequality in the context of nutritional status which helps to revise the existence policy. This article has been well written. However, I have some suggestions which helps to upgrade the concept of the article.

Overall the paper needs English proofreading.

In the first para of introduction, “A systematic review of 128 studies, which

included the period from 1969 and September 30, 2017, shows that the pooled…” this sentence should not be started like that way. Simply state the prevalence and time of the research.

Introduction section is too long to read and understand. Please narrow down it by one and half page with relevant information.

In the section, results of Table 2, please avoid “We see that” this king of wording.

In discussion section, justify the sentence with ref “The possible reasons behind this matter are that educated peoples are habituated on sedentary life styles, and most of them are doing table-chair oriented work (i.e. passing more time in sitting).

Please add some more policy implications that were successful in others developing countries.
