# Supplementary material for: Contribution of sociodemographic determinants in explaining the nutritional gap between the richest-poorest women of Bangladesh: A decomposition approach
Source: PLoS One. 2022 Sep 1;17(9):e0273833. doi: 10.1371/journal.pone.0273833 (PMC9436068; doi:10.1371/journal.pone.0273833)
Supplement: Attachment — Submitted filename: Response to Reviewers.docx [file pone.0273833.s002.docx]

Dear Jing Tian, Academic Editor, PLOS ONE,

Thank you very much for sending me the reviewers comments. In the following table I have provided my response and possible explanations. The first column of the table contains the reviewer’s comments and the second column representing my answers and explanations.

Please let me inform for any queries.

| **Reviewer 1 comments** | **Answers and Explanations** |
| --- | --- |
| Overall comment: This is an interesting piece of research. Inequality exist everywhere in Bangladesh which slows down the progress in every aspect in the country. This research in important to address the inequality in the context of nutritional status which helps to revise the existence policy. This article has been well written. However, I have some suggestions which helps to upgrade the concept of the article. | Thank you very much for your valuable comments. |
| Overall the paper needs English proofreading. | We have checked English carefully to improve the overall quality of language. |
| In the first para of introduction, “A systematic review of 128 studies, which included the period from 1969 and September 30, 2017, shows that the pooled…” this sentence should not be started like that way. Simply state the prevalence and time of the research. | A systematic review shows the pooled prevalence of underweight and overweight were 28% and 17% respectively in the South Asian region during 1969-2017. |
| Introduction section is too long to read and understand. Please narrow down it by one and half page with relevant information. | We have tried to modify the introduction by reducing substantial texts. |
| In the section, results of Table 2, please avoid “We see that” this kind of wording. | We have changed according to your suggestions. |
| In discussion section, justify the sentence with ref “The possible reasons behind this matter are that educated peoples are habituated on sedentary life styles, and most of them are doing table-chair oriented work (i.e. passing more time in sitting). | References are cited. |
| Please add some more policy implications that were successful in others developing countries. | Thank you very much for your suggestions. Policy implications are added in the final paragraph of discussion. |
| **Reviewer 2 comments** | **Answers and Explanations** |
| Overall comment: The applicability of OB decomposition method over other regression-based methods should be discussed.  On many occasions the authors have used the term 'association' inappropriately.  The authors tried to demystify the unequal distribution of BMI between the wealthiest and poorest women. But there is a huge overlap in the distribution of BMI between the two groups and the authors didn’t discuss the limitation of OB decomposition techniques in that context.  There are many grammatical issues that need to be taken care of; discussion section is full of such errors. | We have added the justification of using OB method with reference.  Bivariate analyses are performed to show both percentages of each variable and association between two categorical variables. That’s why we reported associations on many occasions. Hopefully you will be satisfied by our amendments.  Thank you very much for your concern. It is added as a limitation in the revised version.  We have tried our best to address grammatical issues throughout the manuscript. |
| Specific Comments |  |
| Abstract: • Gap in BMI and nutritional gap are not same or similar. BMI is one of the indicators of nutritional status. | This particular part is revised. |
| Background: • The study was not done in context of DBM. Hence, this section is overgeneralised and redundant.  • Suffering from overweight or obese: suffering from overweight or obesity  • For instance, undernutrition (underweight, wasting, stunting) can cause communicable diseases: Explain briefly with reference.  • More specifically, underweight is associated with preterm birth and low birth weight [10, 11], malnourished children: Maternal underweight? Please revise carefully to remove all such ambiguities. | We have deleted the relevant texts of DBM. Hopefully it will fulfil your concern.  Revised accordingly.  Revised with references.  Your observation is right; we have now clearly mentioned maternal underweight. We have also tried to minimize other ambiguities whenever found. |
| Methods: • The continuous measure of BMI is the ultimate outcome variable and that was used to find the contribution of the predictors. Then why did the authors discuss so much about different BMI cut-offs. Needs clarification.  • What is the benefit of using OB decomposition over regression models? Please clarify for the readers.  • Model selection: What was the statistic the authors used for forward selection? Please mention and clarify.  What was the null model?  In section 2.3 authors mentioned that they used contemporary literature review, availability of data and appropriateness techniques for selecting the predictors. | Thank you very much. We have mentioned different categories of BMI to show their prevalence.  Some benefits are added in the statistical analysis part of the methods section.  We have added some statistics (e.g., p-value=0.05 and AIC for determining the best model) necessary for forward selection.  The null model is defined.  We have followed such process to identify potential predictors for the outcome variable. Since not all selected predictors (e.g., food habits, life style, physical exercise) are available in the data set, it is mentioned as a limitation. Only the available variables are used for forward selection. |
| Figures: • Change the title to- “Distribution of BMI according to wealth status”. Association means something different, and the figure is not reporting that. | Revised accordingly. Thank you very much for your suggestions. |
| Tables • Table 1. Take the ‘%(N)’ row beneath the ‘BMI [Mean (SE)]’ row.  • Table 2. The authors presented the proportion of different variables with the corresponding 95% CIs as per the wealth status. They did not report the association. Please consider reviewing the title.  • Table 3. Participants were grouped based on their wealth status. Then what is role of wealth as a predictor variable?  Explain the pooled coefficient of the variable “Wealth”. Interpretation of the terms such as intercept, predicted BMI, total predicted gap is warranted. | Thank you for your comments. Since some variables are continuously measured and analysed through mean and standard error (SE), we indicated them by the symbol ¶. We hope you will kindly accept our modification.  We made some modifications for better representation. Hopefully you will be satisfied.  We provided some explanation in favour of our analysis based on reference of Jann 2008. Please see method section.  Some explanation has given in the results section. |
| Results: • The unexplained part seems to be casting more impact than the explained part of the OB decomposition results. Please explain this and state the limitations related to this unexplained part. | Thank you very much for your valuable comment. We have addressed the explanation with reference in the results section. We also have written this issue as a limitation. |
| Discussion: • From the Kernel density plot we see a huge overlap between the groups. How did the regression model overcome that? How to explain the results in context of such huge overlap?    • “Similarly, mean BMI of the poorest women depicted that they are belonging to normal weight category.”- then why do the authors need to do this decomposition analysis? Just doing a comparison between the overweight/obese and normal weight women would be enough.  • Discussion section should be written focusing on the findings of the study. But here the authors discussed some terms such as physically vigorous situation, burn excess fat of the body, sitting time, contaminated air etc. which they didn’t tested as predictors.  • “However, generally we see the women who spend more time in watching TV or smart phone, does very little or no physical movements.”- very strong statement. Do we have supporting data on this? | Thank you for your comments. But I do not think there is any issue if the density of two groups overlaps. If we look back to the regression line E(y\|x)= alpha + beta*x, it is clear that the regression model works on average rather than individual values and beta coefficient is the difference in mean not the difference in individual values. Though the density overlaps, the mean BMI for richest and poorest classes is clearly different.  Your observation is right. Since the main intention of the study was to analyse the BMI gap between the poorest and the richest categories, we have used the decomposition technique.  Thank you for your comments. Actually we have discussed our findings first. Then we have provided some more explanations based on other papers to make manuscript more comprehensive. Since we didn’t test some of the predictors due to unavailability of data, we have also added them as limitations.  We have modified this part and also added references in favour of our texts. |

With Best Regards

Md Sohel Rana
